# Origin of OXA-23 Variant OXA-239 from a Recently Emerged Lineage of Acinetobacter baumannii International Clone V

**DOI:** 10.1128/mSphere.00801-19

**Published:** 2020-01-08

**Authors:** Lucia Graña-Miraglia, Benjamin A. Evans, Luis E. López-Jácome, Melissa Hernández-Durán, Claudia Adriana Colín-Castro, Patricia Volkow-Fernández, Miguel A. Cevallos, Rafael Franco-Cendejas, Santiago Castillo-Ramírez

**Affiliations:** a Programa de Genómica Evolutiva, Centro de Ciencias Genómicas, Universidad Nacional Autónoma de México, Cuernavaca, Morelos, Mexico; b División de Infectología, Instituto Nacional de Rehabilitación Luis Guillermo Ibarra Ibarra, Mexico City, Mexico; c Instituto Nacional de Cancerología, Mexico City, Mexico; d Norwich Medical School, University of East Anglia, Norfolk, United Kingdom; Escola Paulista de Medicina/Universidade Federal de São Paulo

**Keywords:** evolutionary biology, infectious diseases, population genomics, *Acinetobacter baumannii*, OXA β-lactamases, OXA-239, molecular epidemiology, IC5, OXA, genome analysis, international clones

## Abstract

Over the last few decades, carbapenemase-producing Acinetobacter baumannii has become a major cause of nosocomial infections all over the world. However, the genome identity of lineages of this species in Latin America has not been studied as much as in developed countries. Here, through a population genomics approach considering the whole genomes of 148 isolates (almost 40 from Mexico and Honduras), we describe the recent emergence of the lineage sequence type 758 (ST758), which belongs to the international clone V and has spread out to Canada, Mexico, Honduras, and Colombia. Notably, this lineage was found to coexist with other A. baumannii lineages in hospitals in Mexico and Honduras. Isolates from this lineage show considerable variation in antibiotic resistance profiles, but most of them are resistant to carbapenems. Moreover, we found a variety of acquired oxacillinase (OXA) families within this lineage and tracked the very recent inception, and subsequent horizontal transmission, of the OXA-239 carbapenemase. This work highlights the urgent need to investigate recently emerged lineages of this species in Latin America and elsewhere, as these might harbor novel antibiotic resistance genes.

**IMPORTANCE** A. baumannii is a major cause of nosocomial infections all over the world. Although many isolates from developed countries have been studied in terms of their genome sequence, isolates from Latin America have been much less studied. In this study, using a population genomics approach considering the whole genomes of 148 isolates, we describe the recent emergence of the lineage ST758 endemic to Latin America and the inception of the OXA-239 carbapenemase. Our study highlights the urgent need to investigate recently emerged lineages of this species in Latin America and elsewhere, as these might harbor novel antibiotic resistance genes.

## Abstract (precis)

A. baumannii is a major cause of nosocomial infections all over the world. Although many isolates from developed countries have been studied in terms of their genome sequence, isolates from Latin America have been much less studied. In this study, using a population genomics approach considering the whole genomes of 148 isolates, we describe the recent emergence of the lineage ST758 endemic to Latin America and the inception of the OXA-239 carbapenemase. Our study highlights the urgent need to investigate recently emerged lineages of this species in Latin America and elsewhere, as these might harbor novel antibiotic resistance genes.

## INTRODUCTION

The constant overuse of antibiotics has made many bacterial infections harder to treat, and the global dissemination of antibiotic-resistant bacteria is currently one of the major public health concerns. In this regard, over the last decades, the Gram-negative opportunistic pathogen Acinetobacter baumannii has become a major cause of nosocomial infections with antibiotic-resistant phenotypes all over the world (1). This species consists of several, well-differentiated lineages, and many of them show multidrug resistance (MDR) phenotypes (2). However, the most studied lineages are the three international clones (the European clones I, II, and III) sampled mainly in Asia, Europe, and the United States (3).

The traditional antimicrobial chemotherapy options against A. baumannii are carbapenems, but the constant use of them has been an important factor driving the appearance and dissemination of carbapenem-resistant A. baumannii strains worldwide. This resistance is mainly due to the production of carbapenem-hydrolyzing class D beta-lactamases, a particular group of oxacillinases (OXAs). The intrinsic OXA-51 family of β-lactamases has been found in all isolates of this species and is located in the chromosome, whereas acquired OXA families (OXA-23, OXA-40, OXA-58, OXA-143, and OXA-235) have been found in plasmids and occasionally also in the chromosome (4). Among these acquired families, OXA-23 seems to be the most prevalent around the world (5). OXA-239 is a variant of OXA-23, which has been recently described and only found in Mexico (6, 7).

Whole-genome sequencing (WGS) has become an essential tool to decipher the molecular evolution and phylogeography of bacterial pathogens (8–13). Furthermore, due to the very dynamic genome of A. baumannii, commonly used genotyping strategies (such as multilocus sequence typing [MLST]) do not accurately reveal the genetic relationships of the isolates under study (14). Thus, WGS is the only viable way to establish the true evolutionary relationships of the clones within this species. Additionally, WGS has been of paramount importance to study the transmission dynamics of antibiotic resistance genes (ARGs) in many bacterial populations (11, 15, 16). A considerable amount of information in terms of genomic sequences has been generated for A. baumannii isolates from Europe and the United States, yet very little is known about developing countries. Furthermore, although it is thought that international clones I to III (IC1 to IC3) cause most of the infections in the world, previous studies suggest that this might not be the case for Latin America (3, 17–21). In particular, IC4 and IC5 have been documented in this region (17, 20), and very recently, IC7 was also reported (22).

It is worth mentioning that the genome identity of lineages of this species in Latin America has not been studied as much as in developed countries. To solve this, we recently carried out a few studies. In the first study, we conducted WGS of 8 isolates from Mexico’s National Institute of Oncology (INCAN) collected from 2011 to 2013 (19), whereas in the second study, we sequenced 16 isolates from 5 hospitals in Honduras (23). Here, to extend those two previous data sets and to put together the most extensive data set considering isolates from North America (Canada and Mexico) and Central America (Honduras), we sequenced another 10 isolates from the National Institute of Rehabilitation (INR), which is a tertiary hospital in Mexico City, Mexico. To properly understand the phylogenetic relationships of the Mexican and Honduran isolates, we also included a global data set of publicly available genomes representing many sequence types (STs) and the major international clones. We found an endemic clade (ST758) that recently emerged from within the IC5 and that has considerable variation in terms of antibiotic resistance genes. Moreover, we determined that OXA-239, an OXA-23 variant, very recently emerged within this clade.

## RESULTS AND DISCUSSION

### Novel lineages of A. baumannii.

**FIG 1:**
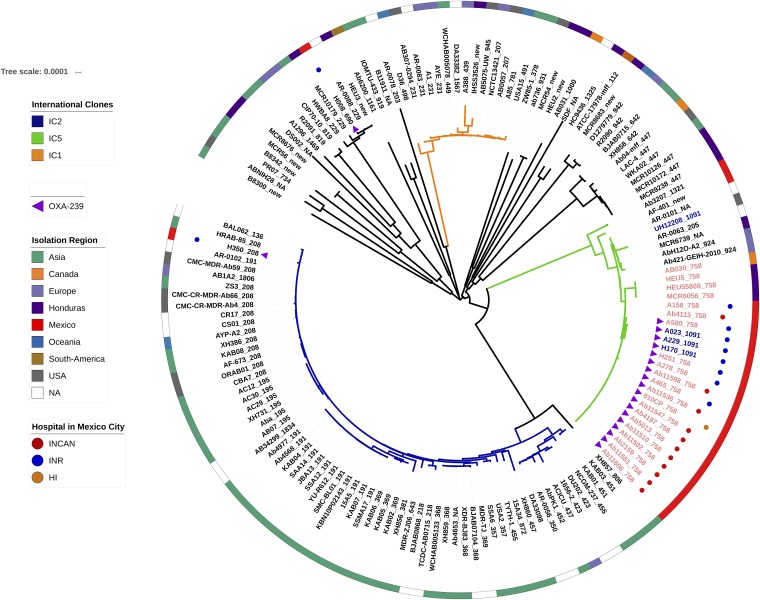
ML phylogeny showing the relationships among all the isolates. The external circle provides the geographic location of the isolates, when available. Colored dots next to some strains denote the Mexican hospitals. International clones I and II are identified by orange and blue branches, respectively, whereas green branches indicate international clone V. Pink isolates show the ST758 lineage, while the three blue isolates (A023, A029, and H170) clustering within the pink isolates belong to ST1091. The presence of the *bla*_oxa-239_ gene is marked with purple triangles. The scale bar gives the number of substitutions per site.

**FIG 2:**
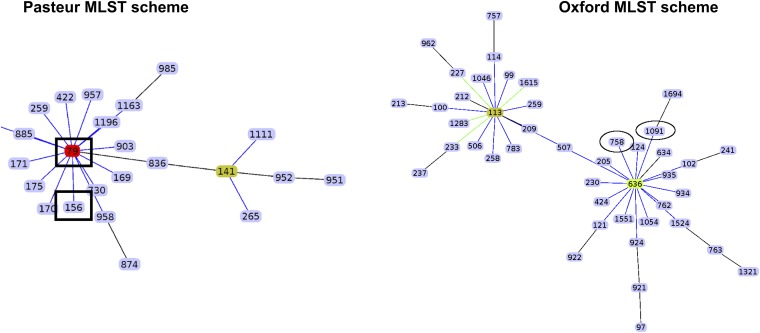
Partial eBURST diagrams for Pasteur and Oxford MLST schemes. Only IC5 is shown for both schemes. Rectangles in the Pasteur scheme show the ST found in some Mexican and Honduran strains (ST156) and the most frequent ST found in South America (ST79, in red), whereas the ovals under the Oxford scheme denote the STs found in some Mexican and Honduran strains (ST758 and ST1091).

Among the almost 150 genomes considered for this study, 38 were either recently published by us or were sequenced in this study (see Table S1 in the supplemental material). These 38 isolates were collected from 2011 to 2016 in 6 hospitals located in 3 different cities in Mexico and Honduras. The maximum likelihood (ML) phylogeny (Fig. 1) shows that 20 of the isolates from Mexico and three from Honduras cluster in one clade (external cycle, Fig. 1). All but 3 isolates from this clade are ST758, as per the Oxford MLST scheme, and these 3 exceptions belong to ST1091, a single-locus variant (SLV) of ST758; this clade clusters with an ST758 Canadian strain (AB030) previously described (24). Both ST1091 and ST758 belong to clonal complex 636 (CC636), which is part of international clone V (IC5) (Fig. 2). According to the PubMLST database, several studies have reported isolates from this ST in Canada (24), Mexico and Honduras (19, 23), and even Colombia (25). Of note, ST758 and ST1091 correspond to ST156 under the MLST Pasteur scheme. Then, given that previous publications considering Latin American isolates (20, 25, 26) have used the MLST Pasteur scheme, we constructed an eBURST diagram using the Pasteur scheme, and a partial view highlighting ST156 is shown in Fig. 2. This analysis shows that ST156 is an SLV of ST79 (Fig. 2) and that both STs are part of the CC79 that corresponds to IC5 (left-hand side, Fig. 2). IC5 has been found in North, Central, and South America and has been considered a Pan-American clone; notably, it very recently has been described in southeastern Europe as well (27). However, there are 11 Honduran isolates and two Mexican isolates that are assigned to other distantly related clades within the tree, demonstrating that several clones coexist in these countries. Regarding the newly sequenced isolates from the INR (blue dots, Fig. 1), we found not only isolates assigned to ST758 but also one isolate (H008) belonging to ST690 and another isolate (H350) assigned to ST208; these three STs are distantly related. We note that a similar pattern applies to some Honduran isolates; for instance, the Mario Catarino Rivas Hospital has more than 5 different STs (some of them are new STs) (Table S1) that are located on distant branches in the phylogeny. Hence, it seems that distantly related lineages could coexist in the very same hospital. The ST758 lineage has very little genetic variation, as judged by the low nucleotide diversity (π = 0.000187), and this could imply a very recent emergence of that clade; therefore, a molecular dating analysis was conducted to obtain an estimate for the emergence of this clade. According to the analysis, this clade emerged very recently in early 2008 (confidence interval, late 2007 to late 2008). This estimate is compatible with the collection dates of the Mexican and Honduran isolates, as none of them were collected before 2010. Taken together, these results imply that several lineages are circulating in hospitals from Mexico and Honduras; the ST758 clade seems to have emerged some 11 years ago and during that period of time has been identified in Central America (Honduras) and North America (Mexico and Canada). On a side note, as noted previously (14), we observed some issues with the MLST genotyping; e.g., ST758 is not a monophyletic group, as three ST1091 isolates (A023, A229 and H170) cluster within the ST758 isolates.

10.1128/mSphere.00801-19.3 TABLE S1 Genomes used for this study. Download Table S1, XLS file, 0.1 MB. Copyright © 2020 Graña-Miraglia et al. 2020 Graña-Miraglia et al. This content is distributed under the terms of the Creative Commons Attribution 4.0 International license.

### Antibiotic resistance profiles.

**FIG 3:**
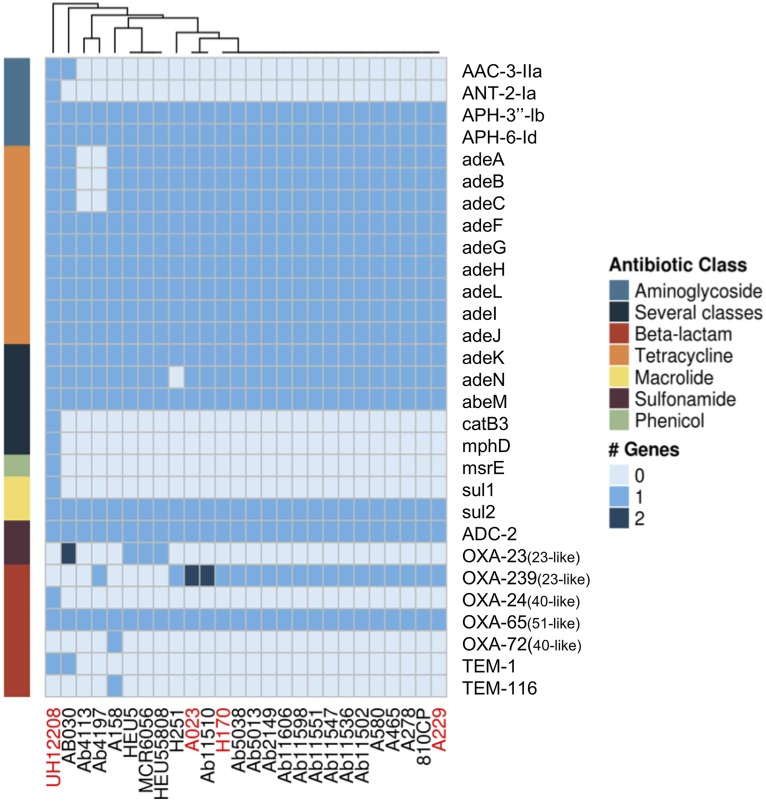
*In silico* prediction of antibiotic resistance genes for some strains from ST758 (in black text) and ST1091 (in red text). A heat map shows how many times a gene is present in the strains considered. The different antibiotic resistance classes are color coded. Strains are grouped according to a hierarchical clustering analysis (cladogram on the top).

Antimicrobial susceptibility tests on the isolates from the INR were conducted, and a high variation in the phenotypic antibiotic resistance profiles among the isolates was noted, but high rates of resistance to many of the antibiotics tested were also found (Table 1). Whereas for the aminoglycosides gentamicin and amikacin we found sensitive, intermediately resistant, and even resistant isolates, for ciprofloxacin, all but one of the isolates were resistant. However, most of these isolates were resistant to doripenem, imipenem, and meropenem, as all but two isolates had MICs higher than 16 mg/liter for all three antibiotics (Table 1). We did not find any isolate resistant to colistin. Clearly, all of these isolates are MDR, as they were resistant to at least one antibiotic in at least three different antibiotic classes. Therefore, although these newly sequenced isolates came from the same hospital (sampled over just 5 years), they present different antibiotic resistance profiles; however, all of the strains were MDR. Then, an *in silico* prediction of the antibiotic resistance gene profiles considering all the isolates within the clade ST758 (Fig. 3) was conducted. All isolates carried the intrinsic *bla*_OXA-51_-like gene (*bla*_OXA-65_ in all cases). Regarding the acquired OXA genes, we noted two isolates with genes from the *bla*_OXA-40_-like gene family; isolate UH12208 contained OXA-24, whereas isolate A158, collected in Mexico, contained OXA-72 (Fig. 3). Most of the isolates (all but three) had genes belonging the *bla*_OXA-23_-like gene family. Interestingly, the most predominant gene of the *bla*_OXA-23_-like gene family was *bla*_OXA-239_, a recently described variant to date only described in Mexico (7). Collectively, our data suggest that these isolates might not only be carbapenem resistant but also MDR showing variation in their resistance profiles. Furthermore, it is very likely that *bla*_OXA-23_-like genes could be functioning as the main carbapenem-resistant determinant.

**TABLE 1:** Antimicrobial susceptibility profiles of the isolates collected from the National Institute of Rehabilitation

Strain	MIC (mg/liter) (resistance breakpoint) for^*a*^:												
	AMK (≥64)	GEN (≥16)	CAZ (≥32)	CEP (≥32)	CIP (≥4)	LVX (≥8)	DOR (≥8)	IPM (≥8)	MEM (≥8)	CST (≥4)	TZP (≥128/4)	MIN (≥16)	AMP-sulbactam (≥32/16)
A023	8	**16**	**>64**	**64**	**64**	**8**	**32**	**64**	**64**	0.125	**>128/4**	0.25	16/8
A158	16	8	**>64**	16	**8**	**8**	**>32**	**32**	**64**	0.5	**128/4**	0.25	8/4
A580	16	8	**64**	**64**	**64**	**8**	**64**	**32**	**32**	0.5	**128/4**	0.25	**64/32**
A229	**64**	8	**>64**	**>64**	**>8**	**8**	**32**	**64**	**32**	1	**>128/4**	0.125	**32/16**
H251	**>64**	**64**	16	**>64**	**>8**	**8**	**>64**	**>64**	**64**	0.5	32/4	0.062	1/0.5
H350	**64**	**64**	**64**	**64**	**64**	**8**	**32**	**32**	**16**	0.25	**128/4**	4	16/8
H170	8	4	**>64**	**>64**	**64**	**8**	**16**	**32**	**32**	0.125	**128/4**	0.062	**32/16**
A465	16	8	**>64**	**>64**	**>8**	**16**	**64**	**64**	**64**	1	**>128/4**	0.25	16/8
H008	16	**32**	**>64**	**64**	**64**	**8**	8	8	8	0.125	**128/4**	0.5	16/8
A278	16	**16**	**>64**	**>64**	**64**	**4**	**32**	**32**	**32**	0.25	**128/4**	0.125	16/8

a Underlining indicates intermediate resistance, and bold type denotes resistance. Intermediate and resistant phenotypes were defined as per the CLSI 2018 guidelines. AMK, amikacin; GEN, gentamicin; CAZ, ceftazidime; CEP, cefepime; CIP, ciprofloxacin; LVX, levofloxacin; DOR, doripenem; IPM, imipenem; MEM, meropenem; CST, colistin; TZP, piperacillin-tazobactam; MIN, minocycline; AMP, ampicillin.

### Natural history of *bla*_OXA-239_.

Then, the evolutionary history of the *bla*_OXA-239_ gene was inferred. We first conducted a BLAST search and did not find any exact match (100% amino acid identity) other than the previously described isolates from Mexico. Notably, none of the isolates having the gene *bla*_OXA-239_ were collected before 2010. Hence, the dates of collection of the isolates having *bla*_OXA-239_ potentially suggest a very recent emergence of this variant. To corroborate this, we conducted another molecular dating analysis using the *bla*_OXA-239_ sequences and their closest *bla*_OXA_ genes. According to this analysis, *bla*_OXA-239_ emerged in early 2010, which completely aligns with the dates of collection of the isolates having this gene. Then, to explore the phylogenetic relationship of *bla*_OXA-239_ with other members of the *bla*_OXA-23_-like gene family, we constructed an ML phylogeny (Fig. S1) considering all the members of the *bla*_OXA-23_-like gene family. The closest sequence to *bla*_OXA-239_ was the *bla*_OXA-469_ gene (Fig. S1) that also has only been described in Mexico, sampled in 2012 (6, 7), and it is associated with ST771. OXA-239 and OXA-469 differ only at the amino acid position 250 (M in OXA-239 and I in OXA-469), and both OXA-239 and OXA-469 had three amino acid substitutions (S109L, D222N, and P225S) compared to OXA-23. These changes have been proposed to be responsible for stabilizing the enzyme and for increasing the enzyme’s substrate profile to include cephalosporins and monobactams, at the expense of a decreased ability to hydrolyze carbapenems (28).

10.1128/mSphere.00801-19.1 FIG S1 ML phylogeny of the *bla*_OXA-23_-like gene family. The inner circle provides the geographic location, whereas the outer circle specifies the OXA type. The colored clade (in orange) is the OXA-239 plus OXA-469 sequences, all found only in Mexico. Blue labels denote OXAs found in A. baumannii isolates, whereas red labels give OXAs present in non-A. baumannii species. Table S2 provides the details (species, accession number, etc.) of all sequences used for this analysis. The scale bar indicates the number of substitutions per site. Download FIG S1, PDF file, 0.1 MB. Copyright © 2020 Graña-Miraglia et al. 2020 Graña-Miraglia et al. This content is distributed under the terms of the Creative Commons Attribution 4.0 International license.

To establish which transposon carries the *bla*_OXA-239_ gene, we located the contig from each strain that carried the *bla*_OXA-23-like_ gene and analyzed the upstream and downstream regions (Fig. S2). The *bla*_OXA-239_ gene is most likely carried on Tn*2008*, as some strains do have IS*Aba1* upstream of the *bla*_OXA-239_ gene. However, this could only be confidently established in the two strains, namely, Ab11510 and 810CP, which were sequenced using the PacBio technology. Remarkably, we did not only find *bla*_OXA-239_ in ST758 isolates but also in two other unrelated lineages, ST690 and ST208 (purple triangles, Fig. 1), indicating that this gene may have been subject to horizontal gene transfer between lineages, as all three of these lineages were found in the same hospital, INR (Fig. 1). Furthermore, for two strains, we recorded two copies of *bla*_OXA-239_ (Fig. 3, A023 and Ab11510); in the case of A023, one of the copies is chromosomally located and the other is in a plasmid, whereas for Ab11510, both copies reside in the chromosome. All in all, these results suggest that the *bla*_OXA-239_ gene has recently emerged in different lineages in Latin America.

10.1128/mSphere.00801-19.2 FIG S2 Contigs that harbor the OXA-23 allele were uploaded to the SimpleSynteny program, which allows visuals to be generated for comparative genome analysis. Eight strains were compared for the sake of clarity, and Ab11510 and 810CP are included as references because they have closed genomes. OXA-239 is colored in green and IS*Aba1* in red. Partial ATP-binding protein can be observed, and in all isolates, DUF-4850 and M13 proteins are continuous to the OXA allele. Most of the assemblies are fragmented next to the OXA-239 allele probably due to the presence of an insertion sequence. Download FIG S2, PDF file, 0.3 MB. Copyright © 2020 Graña-Miraglia et al. 2020 Graña-Miraglia et al. This content is distributed under the terms of the Creative Commons Attribution 4.0 International license.

### Conclusions.

In summary, we have described the recent emergence of an IC5 lineage of A. baumannii that contains a very recently emerged OXA-23-like gene. Importantly, in terms of public health, our study calls attention to the fact that recently emerged lineages should be under surveillance, as these could be reservoirs of new antibiotic resistance genes.

## MATERIALS AND METHODS

### Bacterial isolates, DNA sequencing, and antimicrobial susceptibility testing.

For this study, we have gathered one the most extensive data sets for this species in terms of the diversity of lineages. This data set has 148 genomes and covers more than 50 STs (Table S1). For this study, we sequenced 10 isolates from the INR collected between 2011 and 2015 and sampled from different sources (urine culture, bloodstream, biopsy specimen tissue, endotracheal aspirate, etc.). Samples were isolated and inoculated following standard procedures. The isolates were sequenced using an Illumina MiSeq platform with a 2 × 250-bp configuration. The genome sequencing was conducted at the Instituto Nacional de Medicina Genómica (https://www.inmegen.gob.mx/) in Mexico City. Genomes were assembled with Velvet v1.2.10 (29) and SPAdes v3.11.0 (30), and the best assembly (fewest contigs and largest *N*_50_) was chosen in each case. The antimicrobial susceptibility tests were performed by broth microdilution following the recommendations of CLSI M07-A10 (31) for 14 antibiotics (Table 1).

### Phylogenetic analysis and molecular dating analysis.

A maximum likelihood (ML) phylogeny was performed on the concatenated alignment of 388 nonrecombinant single-gene families (SGFs). Using Roary (32), we identified 766 SGFs whose sequences were at least 90% identical and aligned at least 80% of their lengths. These SGFs were aligned using PRANK (33), and 383 SGFs had evidence of recombination, as per PhiPack (34) (*P* < 0.05), and were discarded. The appropriate evolutionary model for the phylogenetic reconstruction was chosen with ModelTest (https://github.com/ddarriba/modeltest), which was GTR+R+I, and the tree was built with RaxML with 100 bootstrap replicates (35). We also carried out another ML phylogeny of the OXA-23 carbapenemase family. The *bla*_OXA-23_ sequence from strain A85 (NCBI RefSeq accession number NC_025109.1) was used as a query in a BLASTN search against the nucleotide database of the NCBI. We chose hits with more than 90% nucleotide identity (and more than 80% of alignment coverage) since lower parameters recovered other OXA families, and 213 sequences (29 different OXA-23 alleles) were retrieved (Table S2). The sequences were aligned with PRANK, and RaxML was used for the phylogenetic reconstruction. We visualized and annotated the trees using iTOL (36).

10.1128/mSphere.00801-19.4 TABLE S2 Closely related homologous genes of OXA-239. Download Table S2, XLS file, 0.1 MB. Copyright © 2020 Graña-Miraglia et al. 2020 Graña-Miraglia et al. This content is distributed under the terms of the Creative Commons Attribution 4.0 International license.

We ran two molecular dating analyses by means of BEAST 2 (37), one for the concatenated alignment of the nonrecombinant SGF and the other for the *bla*_OXA-239_ gene. The former analysis considered only the ST758 isolates plus the ST1091 isolates that fall within the clade and the two ST924 isolates as an outgroup. In both analyses, the HKY site model with empirical frequencies and a strict clock with a constant population coalescent were set. We ran the analysis for 80,000,000 generations sampling every 40,000 steps. The molecular dating analysis for the *bla*_OXA-239_ gene considered all of the *bla*_OXA-239_ gene sequences plus the *bla*_OXA-469_ gene and *bla*_OXA-255_ gene sequences. This analysis was run for 100,000,000 steps sampling every 10,000 generations. We calibrated the clocks using the dates of collection of the isolates. Both molecular dating analyses were run twice to ensure consistency and made sure that the effective sampling size of the likelihood of the tree and the coalescent model was above 200.

### *In silico* prediction of the antibiotic resistance genes and eBURST analysis.

We carried out BLASTP searches for all of the genes present in the genomes against the Comprehensive Antibiotic Resistance Database (CARD) (38). We chose in each case only the best hit, requiring more than 70% amino acid identity between the query and the target and created a matrix of counts, where we have the genomes in the database and the number of times a certain genetic determinant of resistance was present. For the newly sequenced strains, the absence of a gene was corroborated by aligning the reads against a reference gene sequence to avoid possible negative results due to a fragmented assembly. Resistance profiles of the genomes were visualized using ComplexHeatmap in R. All of the MLST profiles under the Pasteur and the Oxford schemes were downloaded from the PubMLST database (39), and we employed those profiles to construct an eBURST diagram by means of the goeBURST program (40), using the default settings. Contigs that harbor the OXA-23 allele were uploaded to the SimpleSynteny program (41), which allows the generation of visuals for comparative genome analysis. Eight strains were compared for the sake of clarity, and Ab11510 and 810CP were included as references because they have closed genomes (Fig. S2).

### Ethics information.

This study was performed using publicly available genomes and clinical isolates collected at the INR. Regarding the clinical isolates, personal information about the patients is not provided in order to guarantee their anonymity and confidentiality.

### Data availability.

The sequenced genomes were submitted to the NCBI under the BioProject number PRJNA355850, and the accession numbers for the newly sequenced isolates are provided in Table S1.
