# Supplementary material for: Origin of OXA-23 Variant OXA-239 from a Recently Emerged Lineage of Acinetobacter baumannii International Clone V
Source: mSphere. 2020 Jan 8;5(1):e00801-19. doi: 10.1128/mSphere.00801-19 (PMC6952199; doi:10.1128/mSphere.00801-19)
Supplement: FIG S1 [file mSphere.00801-19-sf001.pdf]

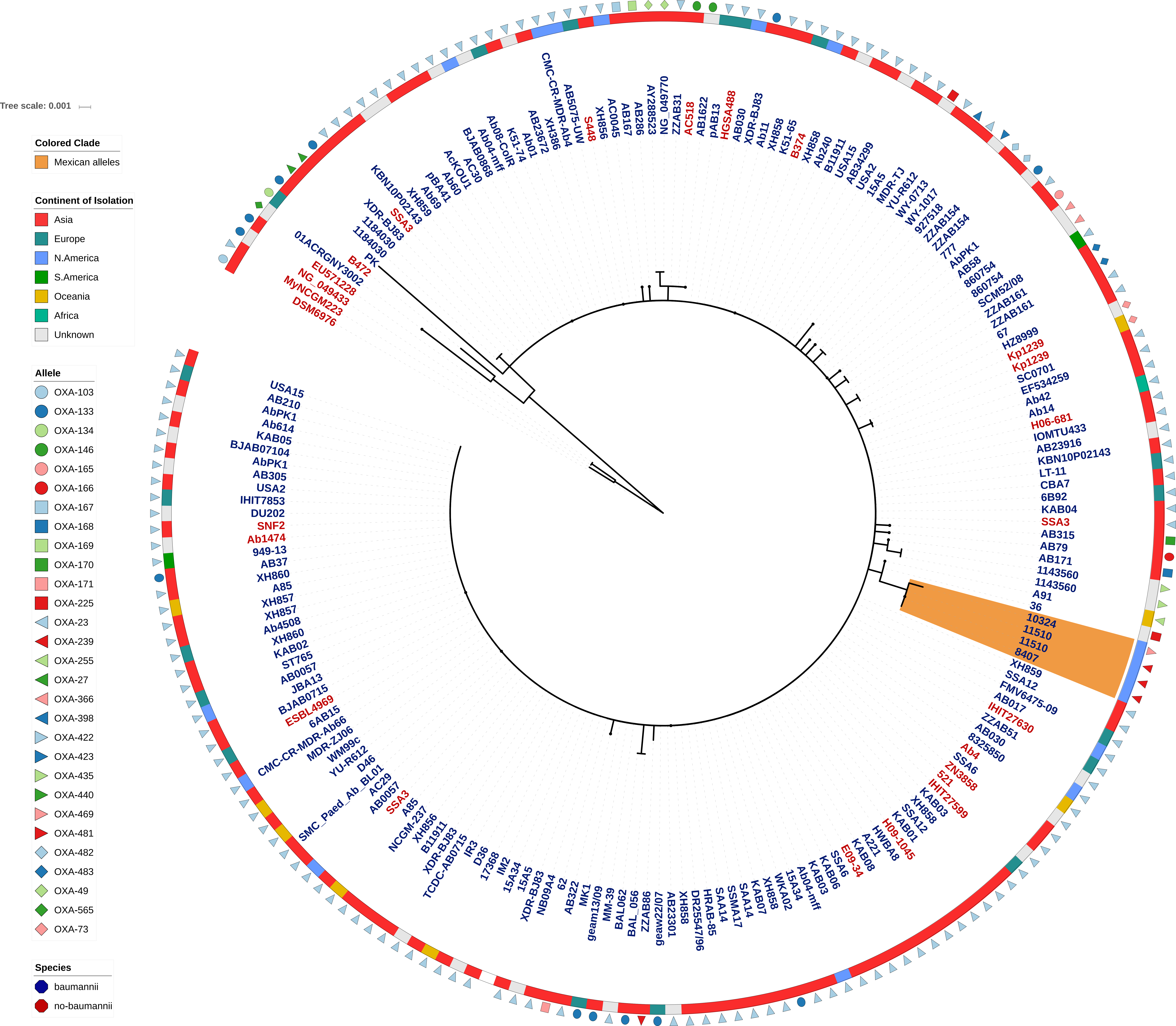

Tree scale: 0.001
Colored Clade
Mexican alleles
Continent of Isolation
Asia
Europe
N.America
S.America
Oceania
Africa
Unknown
Allele
OXA-103
OXA-133
OXA-134
OXA-146
OXA-165
OXA-166
OXA-167
OXA-168
OXA-169
OXA-170
OXA-171
OXA-225
OXA-23
OXA-239
OXA-255
OXA-27
OXA-366
OXA-398
OXA-422
OXA-423
OXA-435
OXA-440
OXA-469
OXA-481
OXA-482
OXA-483
OXA-49
OXA-565
OXA-73
Species
baumannii
no-baumannii
01ACRGNY3002
B472
EU571228
NG_049433
MyNCGM223
DSM6976
PK
1184030
1184030
XDR-BJ83
SSA3
KBN10P02143
XH859
Ab69
pBA41
Ab60
AcKOU1
AC30
BJAB0868
Ab04-mff
Ab08-ColR
K51-74
Ab01
AB23672
XH386
CMC-CR-MDR-Ab4
AB5075-UW
S48
XH856
AC0045
AB167
AB286
AY288523
NG_049770
ZZAB31
AC518
AB1622
pAB13
HGSA488
AB030
XDR-BJ83
Ab11
XH858
K51-65
B374
XH858
Ab240
B11911
USA15
AB34299
USA2
15A5
MDR-TJ
YU-R612
WY-0713
WY-1017
927518
ZZAB154
ZZAB154
777
AbPK1
AB58
860754
860754
SCM52/08
ZZAB161
ZZAB161
67
HZ8999
Kp1239
Kp1239
SC0701
EF534259
Ab42
Ab14
H06-681
IOMTU433
AB23916
KBN10P02143
LT-11
CBA7
6B92
KAB04
SSA3
AB315
AB79
AB171
1143560
1143560
A91
36
10324
11510
11510
8407
XH859
SSA12
FMV6475-09
AB017
IHIT27630
ZZAB51
AB030
8325850
Ab4
SSA6
ZN3858
521
IHIT27599
KAB03
XH858
SSA12
KAB01
H09-1045
HWBA8
A221
KAB08
E09-34
SSA6
KAB06
KAB03
Ab04-mff
WKA02
15A34
XH858
KAB07
SSMA17
SAA14
SAA14
HRAB-85
DR2554/96
XH858
AB23301
geaw22/07
ZZAB86
BAL_056
BAL062
MM-39
geam13/09
MK1
AB322
62
NB09A4
XDR-BJ83
15A5
15A34
IM2
17368
D36
IR3
TCDC-AB0715
XDR-BJ83
B11911
XH856
NCGM-237
A85
SSA3
AB0057
AC29
SMC_Paed_Ab_BL01
D46
YU-R612
WM99c
MDR-ZJ06
CMC-CR-MDR-Ab66
6AB15
ESBL4969
BJAB0715
JBA13
AB0057
ST765
KAB02
XH860
Ab4508
XH857
XH857
A85
XH860
AB37
949-13
Ab1474
SNF2
DU202
IHIT7853
USA2
AB305
AbPK1
BJAB07104
KAB05
Ab614
AbPK1
AB210
USA15
