# Supplementary figures and images for: Origin of OXA-23 Variant OXA-239 from a Recently Emerged Lineage of Acinetobacter baumannii International Clone V
Source: mSphere. 2020 Jan 8;5(1):e00801-19. doi: 10.1128/mSphere.00801-19 (PMC6952199; doi:10.1128/mSphere.00801-19)

Ab11510

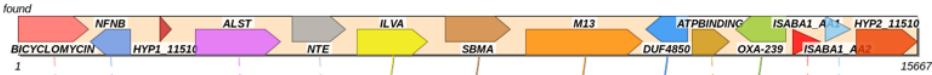

A023

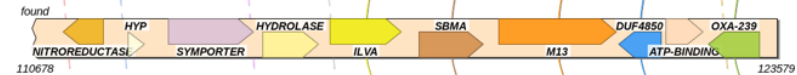

A465

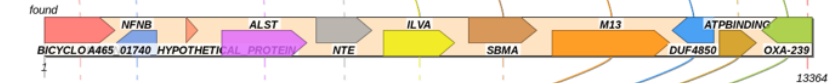

A580

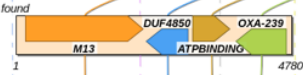

H008

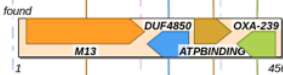

H170

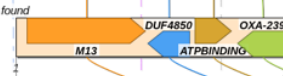

H350

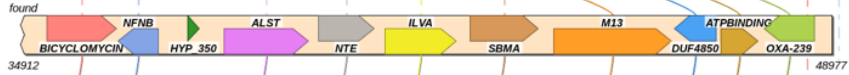

810CP

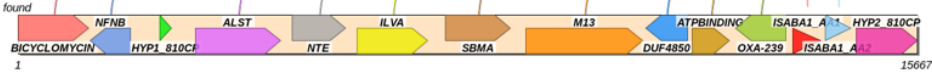

Supplement: FIG S2 [file mSphere.00801-19-sf002.pdf]
